# Autologous pericardial vs. pulmonary artery patches for infant aortic arch reconstruction: clinical and computational morphological outcomes

**DOI:** 10.3389/fcvm.2026.1847610

**Published:** 2026-05-28

**Authors:** Qi Jiang, Chengyi Hui, Wen Zhang, Yifan Zhu, Dian Chen, Renjie Hu, Wei Dong, Jimei Chen, Haibo Zhang

**Affiliations:** 1 Department of Cardiothoracic Surgery, Shanghai Children’s Medical Center, Shanghai Jiaotong University School of Medicine, Shanghai, China; 2 Department of Cardiac Surgery, Guangdong Provincial People’s Hospital (Guangdong Academy of Medical Sciences), Southern Medical University, Guangzhou, China; 3 Department of Cardiothoracic Surgery, Ningbo University Affiliated Women and Children’s Hospital, Ningbo, Zhejiang, China

**Keywords:** aortic coarctation, autologous pericardial patch, autologous pulmonary artery patch, hypoplastic aortic arch, interrupted aortic arch, patch augmentation

## Abstract

**Background:**

The optimal autologous patch material for neonatal and infant aortic arch reconstruction remains debated. We compared the short- and mid-term clinical and morphological outcomes of autologous pericardial patches (PP) vs. pulmonary artery patches (PAP).

**Methods:**

This retrospective, two-center study included 143 patients (≤1 year) undergoing biventricular arch reconstruction for coarctation with arch hypoplasia or interrupted aortic arch (2010–2022). Patients received either PP (*n* = 81) or PAP (*n* = 62). We evaluated clinical outcomes, recoarctation-free survival, and computational arch morphology, including temporal diameter ratios and centerline curvature.

**Results:**

Cardiopulmonary bypass and cross-clamp times were significantly longer with PAP (*P* < 0.001). However, early mortality (overall 4.2%; *P* = 0.698) and 10-year freedom from recoarctation (overall 88.4%; *P* = 0.958) were comparable between the PP and PAP cohorts. Over a 4.0-year median follow-up, an elevated pre-discharge systolic peak velocity >2.48 m/s served as a significant clinical indicator of recoarctation (HR, 7.373; *P* < 0.001). Morphologically, while PAP produced a significantly larger initial proximal arch-to-ascending aorta diameter ratio (*P* < 0.001  ), temporal regression indicated similar mid-term growth trajectories for both patches. Computational centerline analysis showed no significant differences in overall postoperative arch geometry.

**Conclusions:**

Autologous PP and PAP provide comparable clinical and morphological outcomes. Although PAP yields a larger initial arch diameter, longitudinal arch remodeling may be significantly influenced by the native aortic wall. Achieving optimal initial geometric relief is paramount over patch selection to prevent late recoarctation.

## 1 Introduction

In CoA with proximal arch hypoplasia, patch augmentation is not always required; when used, however, it both relieves anastomotic tension and enlarges the hypoplastic segment, reducing the risk of compression of adjacent structures (1, 2). While spontaneous catch-up growth of unrepaired hypoplasia may occur in some cases, it is often inconsistent or incomplete (3); consequently, residual arch abnormality remains a major risk factor for recoarctation and persistent hypertension (4, 5). In IAA, patch augmentation likewise facilitates tension-free reconstruction and enlargement of the proximal arch. In addition, some studies have suggested that acute angulation of the reconstructed aortic arch, termed the “Gothic” arch, which may result from the application of a direct anastomosis technique, is related to late hypertension and subsequent left ventricular dysfunction (6, 7). Patch augmentation enables the reconstruction of an aortic arch with geometry that closely approximates the native physiological configuration (8).

Autologous tissues, primarily pericardial patches (PP) and pulmonary artery patches (PAP), have become preferred materials for pediatric aortic arch reconstruction due to their excellent biocompatibility, absence of immune rejection, and lower risk of infection compared to synthetic patches (8, 9). However, the long-term behavior of these autologous materials differs: PP is typically stabilized with glutaraldehyde, rendering it inert, while the structural evolution and growth potential of fresh PAP under systemic pressure remain a subject of debate (10). Given these distinct histological characteristics and varying institutional preferences, there is a distinct lack of direct comparative studies on their clinical performance (11). To address this literature gap, the present study comprehensively evaluates and compares the short- and mid-term outcomes of neonates and infants undergoing aortic arch reconstruction with PP vs. PAP.

## 2 Materials and methods

### 2.1 Ethics statement

This study was approved by the hospital ethics committee (SCMCIRB-YJ2025013), and the requirement for individual consent was waived.

### 2.2 Patients and grouping

This retrospective study reviewed neonates and infants (≤1year) with CoA and proximal arch hypoplasia or IAA who underwent biventricular repair and aortic arch reconstruction with autologous patches at two centers between 2010 and 2022. All patients in this cohort underwent primary arch reconstruction; those with any prior palliative or cardiac operations were excluded to ensure a homogenous population for patch comparison. Patients with prior arch repair or specific arteriopathies (e.g., Williams syndrome, Takayasu arteritis) were excluded. The cohort was categorized into the autologous pericardial patch (PP) group, or the pulmonary artery patch (PAP) group based on the patch material used.

### 2.3 Surgical technique

All operations were performed via median sternotomy utilizing distal aortic and bicaval venous cannulation, with arch reconstruction achieved under either antegrade cerebral perfusion or deep hypothermic circulatory arrest. After ligation and division of the ductus arteriosus and extensive arch mobilization, the isthmus was transected above and below the ductal insertion, and all ductal tissue was excised. The descending aorta was then anastomosed end-to-side to the posterior distal arch. A longitudinal incision was made along the lesser curvature, extending into the ascending aorta, and connecting to a ventral incision on the descending aorta. Augmentation was completed using either an autologous PP or PAP.

### 2.4 Patch preparation

The PAP was harvested elliptically from the anterior pulmonary trunk, ensuring the preservation of the valve and branch orifices, and was stored in saline until application. The PP was prepared by harvesting autologous pericardium, followed by fixation in 0.6% glutaraldehyde for 8–10 min, and thorough rinsing with saline before use.

### 2.5 Evaluation of aortic arch

Preoperative assessment was performed using transthoracic echocardiography (TTE) and computed tomography angiography (CTA). Postoperative evaluations were conducted before discharge and at 1, 3, and 6 months, and annually thereafter. Routine TTE, primarily utilizing the suprasternal notch view, was employed to assess arch configuration, vessel diameters, and flow velocities. A peak velocity exceeding 2.5 m/s served as the threshold for confirmatory imaging with CTA or magnetic resonance angiography (MRA), with confirmed stenosis requiring reintervention. As the lack of consistent anthropometric data during follow-up precluded the use of vascular Z-scores, the proximal arch-to-ascending aorta diameter ratio was employed to normalize and evaluate patch-related remodeling. The isthmus diameter was explicitly excluded from analysis to avoid confounding effects from anastomotic scarring or residual ductal tissue, which do not reflect the intrinsic impact of the patch. Overall arch morphology was further quantified utilizing the arch height-to-transverse diameter (A/T) ratio and imaging centerline-derived curvature analysis.

### 2.6 Definitions

Preoperative proximal aortic arch hypoplasia was defined as an echocardiography-derived Z-score <−2.0, calculated according to Pettersen et al. (12). Recoarctation was defined as the need for aortic arch reintervention. For morphological assessment, the arch height-to-transverse diameter (A/T) ratio was utilized, where T represents the maximal horizontal distance between the ascending and descending aorta midpoints at the level of the right pulmonary artery, and A is the maximum vertical distance from T to the highest midpoint of the aortic arch.

### 2.7 Statistical and computational analysis

#### 2.7.1 Statistical analysis

Statistical analysis was performed using SPSS 26.0. Continuous variables are expressed as median (interquartile range) and compared using the Mann–Whitney U or Student's t-test, as appropriate. Categorical data are presented as frequencies (percentages) and analyzed via the Chi-square or Fisher's exact test. Receiver operating characteristic (ROC) curves were used to determine the optimal peak velocity cutoff predicting reintervention. Time-to-event outcomes were evaluated using Kaplan–Meier estimates with the log-rank test, and predictors of recoarctation were assessed via Cox proportional hazards models. Statistical significance was defined as *P* < 0.05.

#### 2.7.2 Computational analysis of aortic arch morphology

Quantitative arch morphology and temporal remodeling were analyzed using Python 3.x.

##### 2.7.2.1 Temporal diameter ratio

The proximal arch-to-ascending aorta diameter ratio was tracked over time. Following baseline median assessment, temporal postoperative trends for the PP and PAP cohorts were modeled using logarithmic regression, $y=\beta_{0}+\beta_{1}\ln\;(\chi )$, to capture the non-linear plateauing phase of vascular remodeling. Regression curves (with 95% confidence intervals) were plotted, and overall cohort differences were assessed via Welch's *t*-test to adjust for unequal variances.

##### 2.7.2.2 Centerline curvature analysis

Arch centerlines derived via ImageJ were parameterized from the aortic root to the descending aorta (t=0to1). A spatially weighted 5th-degree polynomial was fitted to the x(t) and y(t) coordinates, with maximal weighting applied to the apex to ensure precise structural representation. Curvature metrics (maximum and mean) were calculated via integral calculus, utilizing the trapezoidal rule for arc length derivation (Supplementary Figure S1).

## 3 Results

A total of 143 patients were included in this study. Baseline characteristics are established in Table 1. Among them, 101 patients (70.6%) were diagnosed with CoA accompanied by proximal arch hypoplasia, while 42 patients (29.4%) were diagnosed with IAA. The median age at the time of operation was 50 days (IQR, 20–87 days), and the median body weight was 4.0 kg (IQR, 3.4–4.5 kg).

**Table 1:** Baseline characteristics according to patch materials.

Variables	Entire group (*n* = 143)	Autologous pulmonary artery patch (*n* = 62)	Autologous pericardial patch (*n* = 81)	*P*-value
Female (%)	46 (32.2)	21 (33.9)	25 (30.9)	0.703
Age at operation (d)	50.0 (20.0,87.0)	53 (29.8,87.3)	47 (15,85.5)	0.200
Weight (kg)	4.0 (3.4,4.5)	4.0 (3.2,4.5)	4.0 (3.5,4.7)	0.267
Aortic coarctation (%)	101 (70.6)	43 (69.4)	58 (71.6)	0.770
Interrupted aortic arch (%)	42 (29.4)	19 (30.6)	23 (28.4)	0.770
Type A (%)	31 (21.7)	14 (22.6)	17 (21.0)	
Type B (%)	9 (6.3)	3 (4.8)	6 (7.4)	
Type C (%)	2 (1.4)	2 (3.2)	0 (0)	
Z score of proximal aortic arch	−3.6 (−4.5,−2.8)	−3.4 (−4.5,−2.8)	−3.7 (−4.6,−2.8)	0.533

Continuous variables are presented as median (interquartile range) and categorical data are presented as *n* (%).

### 3.1 Early results

Early outcomes stratified by patch material are detailed in Table 2. Autologous pericardial patches (PP) and pulmonary artery patches (PAP) were utilized in 81 (56.6%) and 62 (43.4%) patients, respectively. For the overall cohort, the median cardiopulmonary bypass (CPB) time was 113.0 min (IQR, 95.0–141.4), and the aortic cross-clamp (ACC) time was 66.0 min (IQR, 54.3–83.0). Both CPB and ACC times were significantly longer in the PAP cohort (both *P* < 0.001). However, postoperative recovery metrics were comparable between the two groups, with no significant differences observed in intensive care unit length of stay (*P* = 0.182), ventilation duration (*P* = 0.278), the requirement for extracorporeal membrane oxygenation support (*P* = 0.732), or postoperative systolic peak velocity across the reconstructed arch (*P* = 0.887). Early mortality occurred in 6 patients (4.2%), none of which were attributable to aortic arch obstruction (Supplementary Table S1). Crucially, early mortality was not significantly associated with the type of patch material used (*P* = 0.698).

**Table 2:** Early outcomes of patients according to patch materials.

Variables	Entire group (*n* = 143)	Autologous pulmonary artery patch (*n* = 62)	Autologous pericardial patch (*n* = 81)	*P*-value
Cardiopulmonary bypass time (min)	113.0 (95.0, 141.4)	126.0 (106.0, 167.0)	107.0 (92.0, 124.5)	**<0** **.** **001**
Aortic cross-clamp time (min)	66.0 (54.3, 83.0)	76.0 (63.5, 96.8)	64.0 (50.0, 70.5)	**<0**.**001**
Intensive care unit stay (days)	7.5 (6.0, 10.8)	8.0 (5.0, 14.5)	7 (6.0, 9.0)	0.182
Ventilation time (hours)	83.3 (49.9, 132.0)	88.2 (51.7, 131.2)	72.5 (47.0, 122.3)	0.278
Systolic peak velocity (m/s)	1.80 (1.40, 2.08)	1.80 (1.33, 2.08)	1.80 (1.48, 2.10)	0.887
Extracorporeal membrane oxygenation support (%)	9 (6.3)	3 (4.8)	6 (7.4)	0.732
Systolic peak velocity ≥ 2.5 m/s (%)	16 (11.2)	7 (11.3)	9 (11.1)	0.973
Early deaths (%)	6 (4.2)	2 (3.2)	4 (4.9)	0.698

Continuous variables are presented as median (interquartile range) and categorical data are presented as *n* (%).

Bold values indicate statistical significance (*P* < 0.05).

### 3.2 Late results

Median follow-up for the 137 early survivors was 4.0 years (IQR, 2.0–6.5 years), with no late mortality observed.

#### 3.2.1 Reintervention

**Figure 1:**
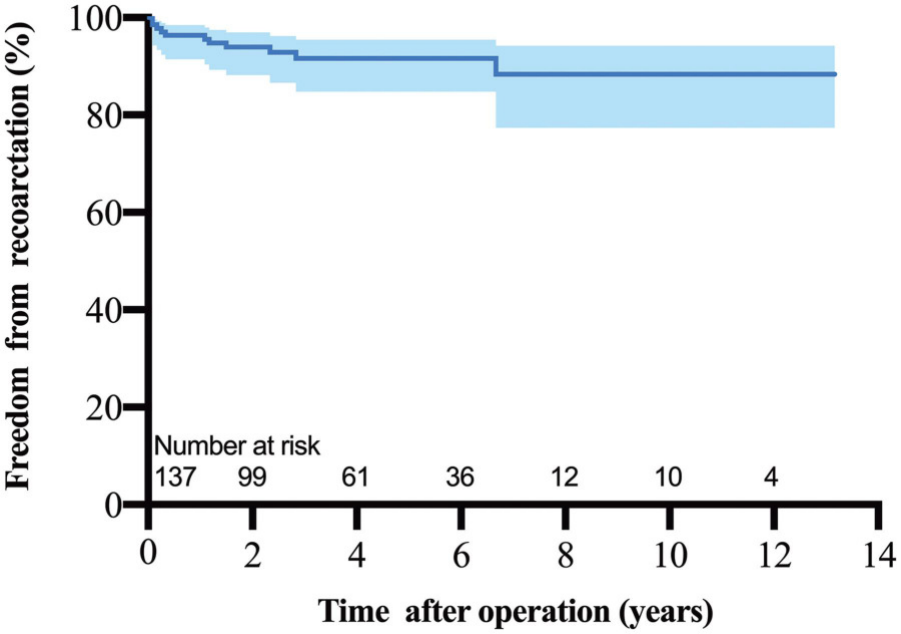
Kaplan–Meier estimates of freedom from recoarctation. The shaded area indicates 95% confidence limits.

**Figure 2:**
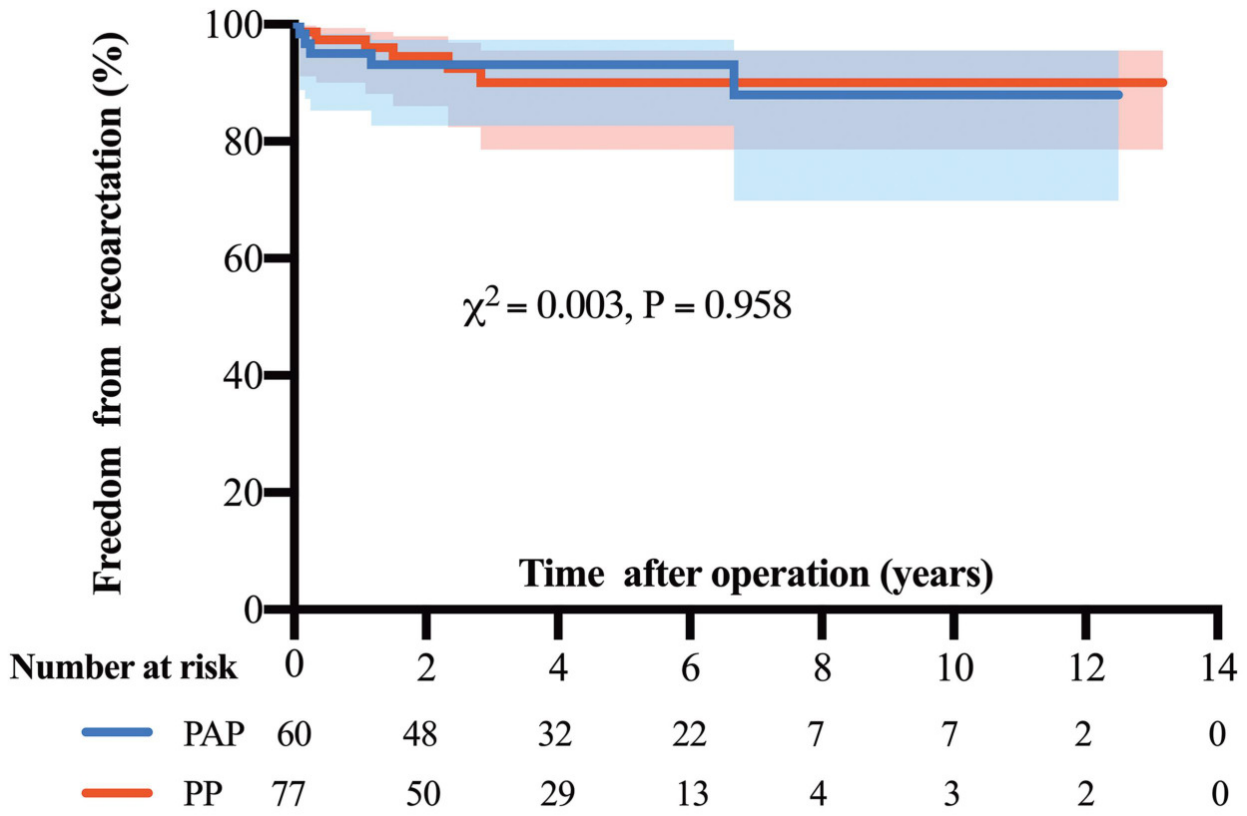
Kaplan–Meier curves for freedom from recoarctation, stratified by patch material: autologous pulmonary artery patch (PAP, blue solid line) and autologous pericardial patch (PP, red solid line). Shaded areas represent 95% confidence intervals. Inter-group differences in time-to-event distributions were assessed using the log-rank test.

Eleven patients (8.0%) required reintervention for recoarctation. This included surgical patch augmentation (*n* = 8; 3 with concomitant intracardiac lesions, 5 with long-segment stenosis) and balloon dilation for localized stenosis (*n* = 3). Overall freedom from recoarctation was 93.9%, 91.6%, and 88.4% at 2, 5, and 10 years, respectively (Figure 1), with no significant difference between the PP and PAP cohorts (*P* = 0.958) (Figure 2). Multivariate Cox regression identified a higher pre-discharge systolic peak velocity across the arch as the most significant clinical marker associated with recoarctation (HR, 7.373; 95% CI, 3.161–17.196; *P* < 0.001) (Table 3). Correspondingly, ROC analysis established a velocity threshold of >2.48 m/s as highly predictive for arch reintervention (AUC, 0.828; 95% CI, 0.683–0.972). Subgroup analysis revealed no significant difference in freedom from recoarctation between CoA and IAA cohorts (*P* = 0.236).

**Table 3:** Factors associated with recoarctation by Cox regression.

Variable	Univariate analysis		Multivariate analysis	
	HR (95% CI)	*P*-value	HR (95% CI)	*P*-value
Interrupted aortic arch (%)	2.888 (0.879–9.484)	0.08	2.545 (0.543–11.923)	0.236
Pre-discharge peak velocity across the aortic arch (m/s)	6.685 (3.192–13.998)	**<0** **.** **001**	7.373 (3.161–17.196)	**<0**.**001**
Autologous pericardial patch (%)	1.031 (0.312–3.413)	0.958		
Cardiopulmonary bypass time (min)	1.013 (1.000–1.026)	0.043	0.985 (0.948–1.023)	0.429
Aortic cross-clamp time (min)	1.029 (1.009–1.049)	0.004	1.046 (0.983–1.113)	0.159
Age (days)	0.984 (0.996–1.003)	0.098	1.000 (0.976–1.025)	0.983
Weight (kg)	0.556 (0.268–1.191)	0.134	0.682 (0.258–1.799)	0.439

HR, hazard ratio; CI, confidence interval. Variables with a *P*-value < 0.15 in the univariate analysis were included in the multivariate Cox regression model.

Bold values indicate statistical significance (*P* < 0.05).

**Figure 3:**
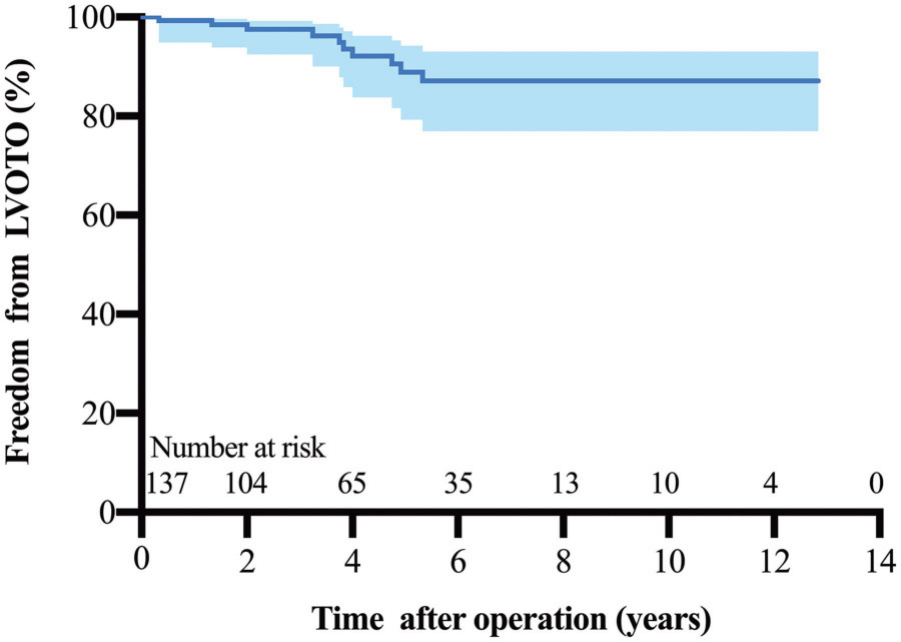
Kaplan–Meier estimates of freedom from left ventricular tract obstruction (LVOTO). The shaded area indicates 95% confidence limits.

Additionally, 10 patients (7.3%) required reoperation for left ventricular outflow tract obstruction (LVOTO). Freedom from LVOTO was 97.5%, 88.9%, and 87.2% at 2, 5, and 10 years, respectively (Figure 3). Although the difference was not statistically significant, there was a trend toward a higher incidence of postoperative LVOTO in the PP group compared with the PAP group (*χ*^2^ = 3.793, *P* = 0.051).

#### 3.2.2 Aneurysm formation and pulmonary artery complications

No aneurysm formed during follow-up. Within the PAP cohort (*n* = 60), 3 patients (5.0%) developed pulmonary artery (PA) stenosis: one in the main PA (Supplementary Figure S2) and two at the branch PA ostia. Two underwent successful balloon dilation for moderate stenosis, while the third (mild-to-moderate) is managed conservatively. All three are currently asymptomatic. No cases of pulmonary valve stenosis or insufficiency were observed. Importantly, no evidence of calcification was observed in the glutaraldehyde-treated pericardial patches on available imaging during the follow-up period.

#### 3.2.3 Morphological and computational analysis of the aortic arch

**Figure 4:**
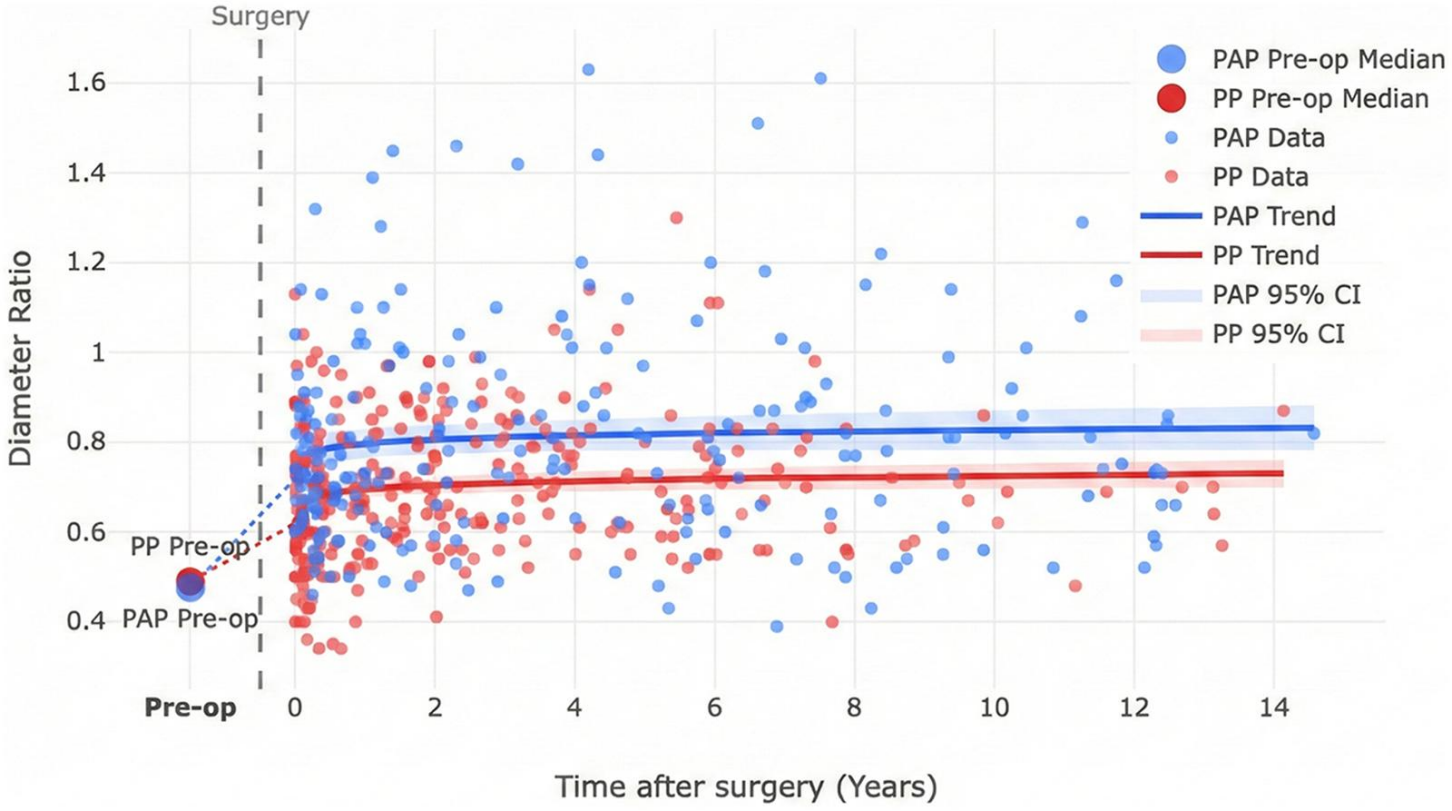
Scatter plots display the diameter ratio over time (years) for the PP (red) and PAP (blue) groups. Pre-operative medians are shown on the left, separated by a vertical dashed line denoting surgery. Solid lines represent the time-adjusted logarithmic regression trends, with shaded areas indicating 95% confidence intervals (CI). The overall post-operative diameter ratio was significantly higher in the PAP group (Mean difference = 0.109, 95% CI: 0.074–0.145; t = 6.09, *P* < 0.001). PP, autologous pericardial patch; PAP, pulmonary artery patch.

The overall postoperative proximal arch diameter ratio was significantly greater in the PAP group compared to the PP group (mean difference = 0.109, 95% CI: 0.074–0.145; *P* < 0.001) (Figure 4). Despite this size discrepancy, temporal regression analysis revealed no significant progressive reduction in diameter ratio over time in the PP cohort relative to the PAP cohort.

Computational morphological analysis revealed no significant differences in overall arch geometry between the PAP and PP cohorts. Comparable outcomes were observed for the median A/T ratio (0.55 [IQR, 0.51–0.61] vs. 0.55 [IQR, 0.51–0.62]; *P* = 0.719), median apex curvature (0.032 mm⁻^1^ vs. 0.037 mm⁻^1^; *P* = 0.346), median mean curvature (0.023 mm⁻^1^ vs. 0.021 mm⁻^1^; *P* = 0.366), and chord length-to-arc length ratio (0.667 ± 0.068 vs. 0.659 ± 0.057; *P* = 0.552).

## 4 Discussion

### 4.1 Patch selection and surgical outcomes

The optimal choice of patch material for aortic arch reconstruction in neonates and infants remains a subject of ongoing debate (11, 13–16). To achieve complete relief of arch stenosis, avoid compression of adjacent structures, and maintain physiological arch geometry, both participating centers routinely performed patch augmentation. Given the limited availability of homografts, we primarily utilized autologous materials: the autologous pericardial patch (PP) and the autologous pulmonary artery patch (PAP).

PP is favored for its availability, ease of harvest, and good tissue compatibility. Though concerns regarding long-term durability persist (17), recent studies have demonstrated its sufficient tensile strength, supporting its use for arch reconstruction (13). Crucially, all PPs in this study were glutaraldehyde-fixed, which likely contributed to the absence of postoperative aneurysm formation, contrasting with concerns associated with fresh pericardium (18). The PAP provides tissue similar to the native aortic wall, offering potential for adaptive growth and minimizing aneurysm risk (6, 19, 20), though harvesting may potentially cause pulmonary artery trauma.

Both patch materials yielded satisfactory clinical outcomes in this study. The PAP group demonstrated a low incidence of recoarctation (8.3%) and mortality (3.2%), with no aneurysms detected during follow-up. The PP group showed a mortality rate of 4.9% and a recoarctation rate of 7.8%, with no significant difference compared to the PAP group. Furthermore, the CPB and ACC times were significantly shorter in the PP group than in the PAP group, reflecting a simpler operative technique. Unlike earlier studies that rarely noted this complication (21), three cases (5.0%) of postoperative pulmonary artery stenosis occurred in our PAP cohort, likely related to the patch harvesting procedure. The observed PA stenosis in the PAP group, despite reconstruction of the donor site with an autologous pericardial patch, may be attributed to restrictive patch sizing or localized distortion of the pulmonary trunk geometry during suturing. This occurrence underscores that while harvesting autologous pulmonary tissue is viable, technical precision—specifically regarding the sizing of the supplemental pericardial patch and careful suture placement—is essential to preserve the native architecture of the pulmonary trunk and mitigate the risk of late stenosis.

### 4.2 Key predictors of recoarctation

Regardless of the patch material utilized, multivariate Cox regression analysis identified a higher systolic peak velocity across the arch prior to discharge as a strong, independent predictor of late recoarctation. Consistent with previous reports (22), an inadequate initial relief of the stenosis or an undersized postoperative isthmus is inextricably linked to a higher risk of reintervention. This strongly suggests that sufficient arch enlargement and complete resection of the ductal tissue at the index operation are more critical than patch selection in preventing late obstruction.

### 4.3 Influence of patch material on arch configuration

This study also examined whether the two autologous patch types influence the postoperative configuration of the aortic arch. Early postoperatively, the proximal arch diameter was significantly larger in the PAP cohort compared with the PP cohort. We hypothesize this dimensional discrepancy is multifactorial, driven by distinct differences in tissue biomechanics, inherent geometry, and surgical handling. Unlike glutaraldehyde-fixed pericardium, which becomes stiff and non-compliant due to collagen cross-linking, fresh autologous pulmonary artery tissue retains its native elastin network, allowing it to readily distend under high systemic pressures. Additionally, the PAP is harvested as a naturally curved, three-dimensional segment, inherently providing a more voluminous cross-sectional area than a flat pericardial sheet. Finally, accommodating the concave edges of the PAP dictates a wider, flared anastomotic profile, further contributing to a broader initial arch diameter.

However, over time, the PP group showed no significant reduction in this diameter ratio compared with the PAP group, suggesting that the glutaraldehyde-fixed pericardium maintained its structural stability during growth. These findings indicate that mid-term arch growth is primarily attributable to the remaining native aortic wall rather than the patch itself.

Clinically, these morphological findings suggest that while PAP may be advantageous in cases of hypoplasia requiring maximum initial diameter, the eventual geometric outcome is largely independent of patch material. This allows for surgeon discretion based on patch availability and technical familiarity without compromising long-term arch configuration.

Moreover, computational centerline analysis demonstrated no significant differences in overall arch geometry (height-to-width ratio and curvature) between the two groups. This further supports the conclusion that patch material does not substantially influence the reconstructed arch configuration.

### 4.4 Limitations

This study is subject to several limitations. First, its retrospective, non-randomized nature introduces inherent selection bias, as patch selection was influenced by institutional and surgeon preference. Second, the inconsistent availability of somatic growth parameters during follow-up prevented the use of standardized Z-scores for evaluating arch dimensions. Consequently, the primary limitation remains the lack of longitudinal Z-score data, and an internal ratio (proximal arch-to-ascending aorta diameter) was utilized as a surrogate marker for vascular remodeling. Additionally, as cross-sectional imaging was not performed systematically for all patients, direct assessment of patch wall thickness and mechanical behavior over time was not feasible. Third, while the cohort is sizable for this specific pathology, it may lack the statistical power to definitively capture rare late complications like aneurysm formation. Furthermore, while specific patch surface area measurements were not systematically recorded, patches were tailored to achieve a reconstruction diameter matching the native ascending aorta. The influence of initial patch dimensions on localized hemodynamics remains a factor for future prospective evaluation. Fourth, the median follow-up of 4.0 years represents early-to-mid-term outcomes; tracking these patients into adolescence is essential to fully elucidate the long-term structural evolution and genuine growth potential of both patch materials. Additionally, our computational analysis carries methodological constraints: manual arch centerline tracing is susceptible to inter-observer variability, and polynomial curve fitting serves as a simplified mathematical approximation of complex *in vivo* arch biomechanics. Finally, granular anatomical details, such as the specific length of the hypoplastic segment or interruption gap, were not available for analysis and may influence the required patch geometry.

## 5 Conclusions

Autologous PP and PAP provide comparable clinical and morphological outcomes for neonatal and infant aortic arch reconstruction. While PAP yields a larger initial arch diameter, both materials exhibit similar mid-term growth trajectories, suggesting that the mid-term remodeling pattern may be significantly influenced by the native aortic wall rather than the patch material itself. An elevated pre-discharge systolic peak velocity (>2.48 m/s) independently predicts late recoarctation, underscoring that optimal geometric relief of the obstruction at the index operation is paramount over patch selection.

## Data Availability

The original contributions presented in the study are included in the article/Supplementary Material, further inquiries can be directed to the corresponding authors.
