# Supplementary material for: Autologous pericardial vs. pulmonary artery patches for infant aortic arch reconstruction: clinical and computational morphological outcomes
Source: Front Cardiovasc Med. 2026 May 28;13:1847610. doi: 10.3389/fcvm.2026.1847610 (PMC13254060; doi:10.3389/fcvm.2026.1847610)
Supplement: Supplementary file 1 [file Datasheet1.docx]

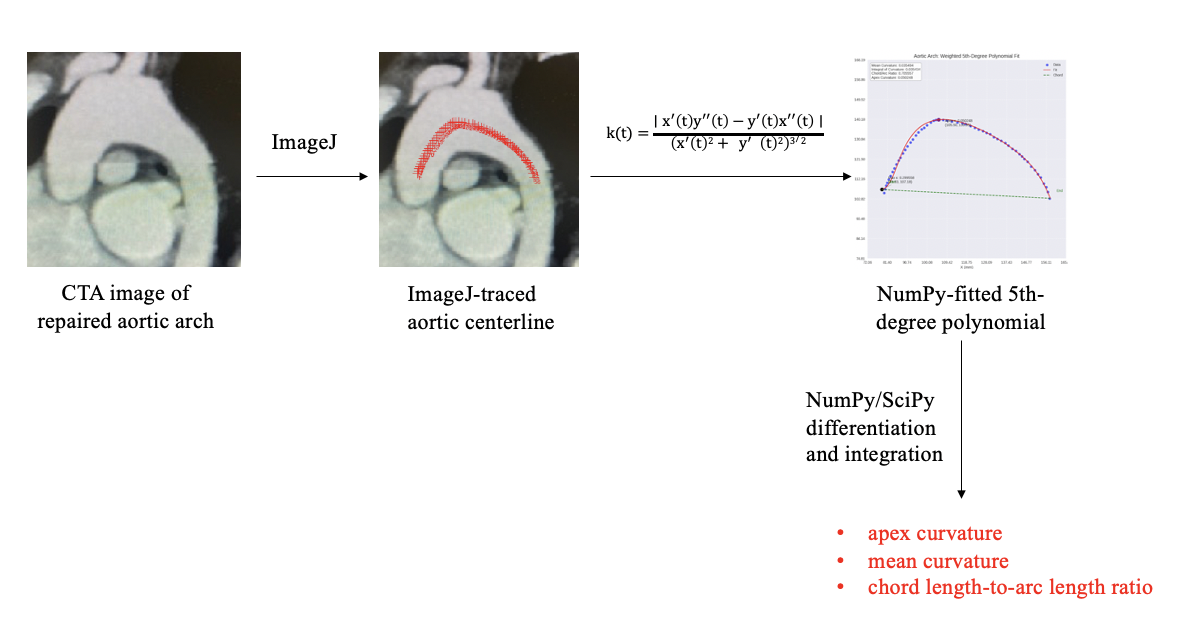


Supplementary Figure 1. Workflow of centerline curvature analysis


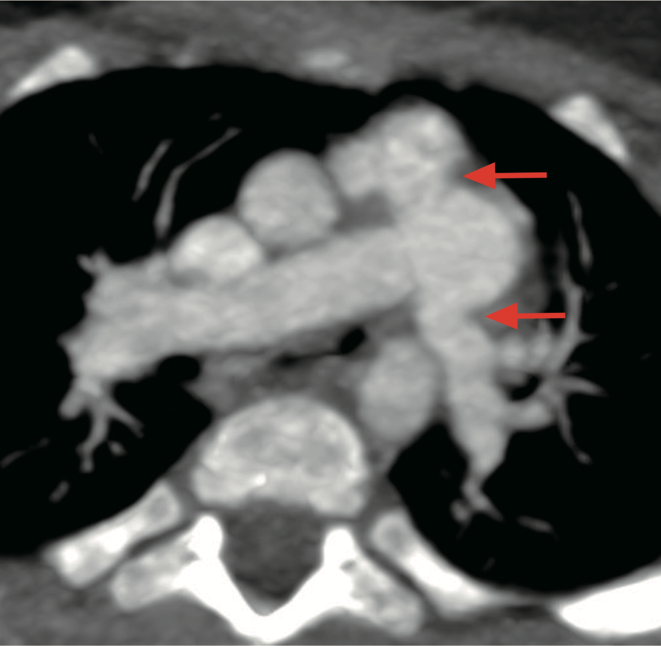


Supplementary Figure 2. Postoperative computed tomographic angiography showing pulmonary artery stenosis in PAP group. The stenosis sites are labeled by red arrows.

Supplementary Table S1. Causes of early mortality

| Causes of mortality | Surgical Procedure |
| --- | --- |
| Myocardial dysfunction (n=4) | - IAA repair + VSD closure + ASD closure (n=2) - Hypoplastic arch repair + VSD closure + ASD closure (n=2) |
| Pulmonary hypertension crisis (n=1) | - IAA repair + VSD closure + PFO closure |
| Multi-organ failure (n=1) | - Hypoplastic arch repair + DORV repair + ASD closure |

ASD: atrial septal defect; DORV: double outlet right ventricle; IAA: interrupted aortic arch; PFO: Patent foramen ovale; VSD: ventricular septal defect.
